# Phage–host interplay: examples from tailed phages and Gram-negative bacterial pathogens

**DOI:** 10.3389/fmicb.2014.00442

**Published:** 2014-08-20

**Authors:** Soraya Chaturongakul, Puey Ounjai

**Affiliations:** ^1^Department of Microbiology, Faculty of Science, Mahidol UniversityBangkok, Thailand; ^2^Center for Emerging Bacterial Infections, Faculty of Science, Mahidol UniversityBangkok, Thailand; ^3^Department of Biology, Faculty of Science, Mahidol UniversityBangkok, Thailand

**Keywords:** bacteriophage, host–phage interaction, phage resistance mechanism, host–phage dynamics, microbial community

## Abstract

Complex interactions between bacteriophages and their bacterial hosts play significant roles in shaping the structure of environmental microbial communities, not only by genetic transduction but also by modification of bacterial gene expression patterns. Survival of phages solely depends on their ability to infect their bacterial hosts, most importantly during phage entry. Successful dynamic adaptation of bacteriophages when facing selective pressures, such as host adaptation and resistance, dictates their abundance and diversification. Co-evolution of the phage tail fibers and bacterial receptors determine bacterial host ranges, mechanisms of phage entry, and other infection parameters. This review summarizes the current knowledge about the physical interactions between tailed bacteriophages and bacterial pathogens (e.g., *Salmonella enterica* and *Pseudomonas aeruginosa*) and the influences of the phage on host gene expression. Understanding these interactions can offer insights into phage–host dynamics and suggest novel strategies for the design of bacterial pathogen biological controls.

## INTRODUCTION

Bacteriophages (or phages) are the most abundant and most diversified microorganisms on Earth. Acting as obligate bacterial predators, phage can be found in all reservoirs populated by bacterial hosts, e.g., in soil (Marsh and Wellington, 1994; Kimura et al., 2008), in aquatic environments (Bergh et al., 1989; Suttle, 2007), and even in the human gut (Breitbart et al., 2003; Mills et al., 2013). The evolutionary success of bacteriophages, estimated at approximately an order of magnitude and up to a 25-fold greater abundance in comparison with their bacterial hosts (Fuhrman, 1999), indicates remarkable dynamic adaptability of phages in nature.

In natural habitats, phages and bacteria are in a constant arms race that proceeds in continuous cycles of co-evolution (Thingstad and Lignell, 1997). As bacteria develop mechanisms to prevent phage infection, e.g., bacterial receptor modification and degradation of invading phage DNA (Labrie et al., 2010), phages can circumvent the resistance and evolve mechanisms to target such resistant bacteria (Samson et al., 2013). This arms race continues and become one of the major forces to both expand global genetic diversity and maintain balance within microbial communities.

Previous reviews of phage–bacteria interaction mainly focused on model organisms such as *Escherichia coli* phages (or coliphages) and bacterial CRISPR/Cas immunity systems against phage genome replication. The focal points of this review, however, are physical interactions between tailed phages and other Gram-negative bacterial pathogens, specifically *Salmonella enterica* and *Pseudomonas aeruginosa*, and the influences of phage–host interactions on the gene expression of these clinically important bacteria and, more generally, on microbial diversity.

## PHYSICAL INTERACTIONS BETWEEN TAILED PHAGES AND THEIR HOSTS

**FIGURE 1:**
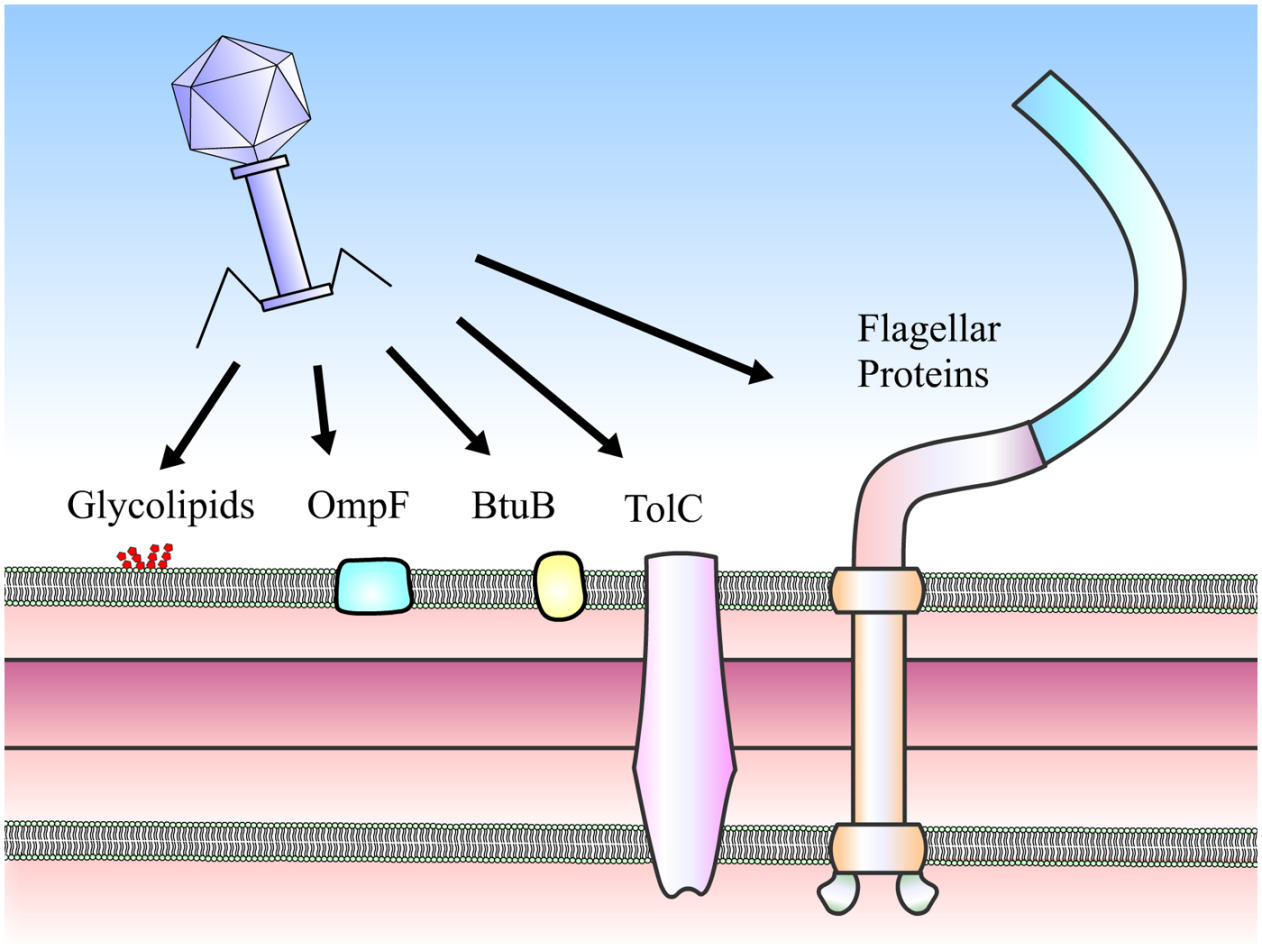
**Receptors of *Salmonella* phages.** Phages can use a number of cell surface moieties as receptors, including glycolipids (O- and Vi-antigens), integral membrane proteins (e.g., OmpF, BtuB, and TolC) and flagella proteins (FliC, FljB, and FliK).

To initiate infection, phages need to adsorb to the host surfaces, penetrate cell walls and inject genetic materials into the host. Mechanisms used to initiate the connection to bacterial hosts prior to phage genome injection are referred to as tails. Ackermann and Prangishvili (2012) demonstrates that over ninety percent of approximately 6,200 phages examined by electron microscopy (EM) are tailed phages in the *Caudovirales* order (i.e., siphophages, myophages, and podophages). As the name implies, the adsorption machinery dedicated for specific host recognition is localized at the tail-end, varying from a simple tail tip to a complex base plate. Such mechanisms appear to be well-correlated with host adsorption strategies (Veesler and Cambillau, 2011; Fokine and Rossmann, 2014). Some tail structures can be quite complex and include extra-structures including tail spikes and tail fibers (Fokine and Rossmann, 2014). This adsorption machinery is the most rapidly evolving part of the tailed phage genome (Hendrix et al., 1999). Tail proteins of these phages are diverse and capable of recognizing almost every host surface component (**Figure 1**), including surface proteins, polysaccharides and lipopolysaccharides (LPS; Lindberg, 1973).

The interactions between phages and hosts occur between phage tail proteins and bacterial receptors, which are proteins or LPS. These interactions determine host specificity and range. Known receptors (**Table 1**) of *Salmonella* phages are flagella proteins (e.g., FliC and FljB, Shin et al., 2012; and FliK, Choi et al., 2013), outer membrane porin or osmoregulatory protein OmpC (Ho and Slauch, 2001; Marti et al., 2013), outer membrane protein for vitamin B_12_ uptake BtuB (Kim and Ryu, 2011), outer membrane protein for drug eﬄux pump TolC (Ricci and Piddock, 2010), outer membrane transport protein FhuA (Casjens et al., 2005), O-antigen of LPS (Shin et al., 2012), and Vi capsular antigen (Pickard et al., 2010). On the other hand, known phage receptors in *P. aeruginosa* are type IV pili (Heo et al., 2007; Bae and Cho, 2013) and O-antigen of LPS (Le et al., 2013).

**Table 1:** Specific host receptors for *Salmonella* and* P. aeruginosa* phages.

	Specific host receptors	Reference
*S. enterica*	*Flagellar proteins*	
	FliC and FljB	Shin et al. (2012)
	FliK	Choi et al. (2013)
	*Outermembrane proteins*	
	OmpC	Ho and Slauch (2001), Marti et al. (2013)
	BtuB	Kim and Ryu (2011)
	TolC	Ricci and Piddock (2010)
	FhuA	Casjens et al. (2005)
	*Surface antigens*	
	O-antigen	Shin et al. (2012)
	Vi-antigen	Pickard et al. (2010)
*P. aeruginosa*	*Surface antigens*	
	O-antigen	Le et al. (2013)
	Vi-antigen	Temple et al. (1986), Hanlon et al. (2001)
	*Type IV pili*	
	PilA	Bae and Cho (2013), Heo et al. (2007)

The protein–protein interactions between phage tail fiber proteins and bacterial flagella filaments are specific and strong. Such flagellatropic phages adsorb to the bacterial hosts via flagellin proteins, i.e., phase 1 antigen FliC, phase 2 antigen FljB, or flagella hook FliK (Shin et al., 2012; Choi et al., 2013). The preference for each flagella receptor varies among phages. *Salmonella* siphophages can use only FliC or both FliC and FljB as receptors (Shin et al., 2012). The latter is more prevalent as variety in host receptors leads to wider possible host ranges. In addition to being flagellotropic, host adsorption in some phages is also dependent upon motility and the directional rotation of the flagella themselves. For instance, *Salmonella* phage iEPS5 requires adsorption to either FliC or FljB flagellin and successful host infection only occurs if the flagella are rotating counter-clockwise in the presence of flagella torque protein MotA and hook protein FliK (Choi et al., 2013). The evidence also suggests that, alongside the adsorption step, injection of iEPS5 DNA is also flagella-dependent, possibly through the flagellin channel (Choi et al., 2013). In addition to adsorption through flagella, phages can also attach through the pili. Specifically, *P. aeruginosa* phage MPK7 and M22 utilize type IV pili as their receptors. *P. aeruginosa* hosts lacking *pilA* gene are resistant to infection by these phages (Heo et al., 2007; Bae and Cho, 2013).

Besides motility apparatus such as flagella and pili, outer membrane proteins are also targets for phage adsorption. OmpC porin is used as a receptor by *Salmonella* Gifsy and T4-like phages (Ho and Slauch, 2001; Marti et al., 2013), while vitamin B_12_ uptake protein BtuB is used by T5-like phages (Kim and Ryu, 2011). Although resistance to BtuB-targeting phages can develop in *Salmonella*, the trait is not heritable and bacterial daughter cells can revert and become susceptible to these phages again (Kim and Ryu, 2011). Additionally, a conserved innate eﬄux pump TolC is used by *Salmonella* phage as a receptor (Ricci and Piddock, 2010). TolC has also been shown to be a colonization factor of *S. Typhimurium*, in which a *tolC* null strain is virulence attenuated (Webber et al., 2009). Therefore, application of TolC targeting phages is expected to have dual advantages, as the phages themselves can directly infect the bacterial host and can also drive the emergence of TolC-deficient *Salmonella*, which is incapable of colonization and incapable of spreading infection. Like coliphages, *Salmonella* phages use ferrichrome transport protein FhuA as a receptor (Casjens et al., 2005).

Bacteriophages also use surface antigens such as O-and Vi-antigens as receptors. In both *S. enterica* and *P. aeruginosa*, O-antigens of LPS are phage receptors. In *Salmonella*, tail spike proteins of the typical siphophages or podophages not only recognize but also hydrolyze the O-antigen, allowing the phage to penetrate through the 100-nm O-antigen layer during infection (Andres et al., 2012). Siphophage SSU5, however, uses core oligosaccharides of LPS as receptors (Kim et al., 2014), making SSU5 a beneficial addition to the phage cocktail against insensitive *Salmonella* populations capable of O-antigen glucosylation (Kim and Ryu, 2012). Other than O-antigens, Vi capsular antigens of *S. Typhi* are also targets for recognition by phage tail protein, specifically at a conserved acyl esterase domain (Pickard et al., 2010). On the other hand, phages of *P. aeruginosa* recognize O-antigens of LPS using tail fibers (Le et al., 2013). Interestingly, extracellular matrix-like structures such as alginate glycopolysaccharide layers are also found in *Pseudomonas* spp. The role of these layers is to physically alter phage accessibility. Phage F116 of* Pseudomonas* can produce alginate lyase which dissolves the alginate layer and facilitates penetration and dispersion of phages in such a matrix (Hanlon et al., 2001). However, alteration in alginate production renders *Pseudomonas* insensitive to phage2 and φPLS-I, thus revealing the role of alginate in adhesion (Temple et al., 1986).

It is important to note that interactions between phages and host bacteria are not exclusive to single types of phage protein-receptor recognition. Shin et al. (2012) demonstrated cross-infection and cross-resistance among phages recognizing different targets on *S. enterica,* e.g., bacterial hosts that are resistant to flagellatropic phages are sensitive to phages targeting BtuB and LPS. Conversely, due to possible interactions between BtuB and LPS targets, bacterial strains that are resistant to BtuB-targeting and LPS-targeting phages are susceptible to flagellatropic phages (Shin et al., 2012). According to the “killing the winner” hypothesis (Thingstad and Lignell, 1997), cross-infection by different types of phages naturally limits the abundance of successful strains and thereby increases bacterial diversity. Although a vast variations of phage tails and other adsorption structures have been observed by EM (Ackermann and Prangishvili, 2012), the X-ray structures of phage tail proteins including Dit and gp27-like proteins from various phages demonstrate remarkable structural similarities which suggest a common evolutionary origin for phage tail proteins (Veesler and Cambillau, 2011).

## PHAGE–BACTERIA: GENETIC GIVE-AND-TAKE

Rapid reciprocal evolutionary competition between bacteria and phages (and even among phages with common bacterial hosts in a shared environment) creates high selective pressure, forcing diversification of the attachment-related structures and the emergence of various phage infection tactics (as reviewed in Veesler and Cambillau, 2011). However, success in infection is apparently not sufficient for phage survival in nature. As an obligate parasite, phages are dependent upon the survival of their host population. Thus, the availability of hosts is at least as important in determining the environmental fitness of phages. Phages must also modulate the gene expression of host cells to prevent superinfection by other phages.

In a lysogenic life cycle, phage integrates its genome and replicates along with its host. Not only the host survival is sustained, the presence of prophages has also been repeatedly shown to be beneficial. Prophages can provide their host population with immunity against secondary infection or superinfection by other incoming phages. In lysogenic *S. enterica,* expression of podophage P22-encoded proteins SieA and SieB exclude superinfection through degradation of the superinfecting phage genome (Susskind et al., 1974a) and induce lysis of superinfected host cells, protecting the whole host population (Susskind et al., 1974b). Similarly, the expression of siphophage HK97-encoded protein gp15 protein excludes superinfection by other HK97 phages via inhibition of phage genome entry (Cumby et al., 2012).

Beyond superinfection exclusion, prophages can also enhance the fitness of their hosts in the environment. A recent study in *P. aeruginosa* PAO1 has shown that infection with LPS-targeting E79 myophage for 4 days gave rise to E79-immune variants that, while exhibiting slower growth than control PAO1 and impairment of twitching, swimming and swarming abilities, produced more virulence factor pyocyanin and were less frequently internalized by J774 macrophages (Hosseinidoust et al., 2013). In addition, several lines of evidence demonstrate extensive genetic circulation amongst phage and host communities. In fact, approximately 20% of the extant genetic content of a given bacterial species are acquired (Gogarten et al., 2002). These acquired genetic materials include so-called mobile genetic elements, including several bacterial pathogenicity islands which can potentially be horizontally transferred across species boundaries. Some phage-related chromosomal islands use phages as transduction vectors (Novick et al., 2010). This phage-mediated horizontal transfer of genetic material is of extreme importance as it acts as an active driving force behind bacterial evolution. Classic examples are the *P. aeruginosa* gigantic bacteriocin called R- and F-type pyocins which are utilized to combat other microbes in the community (Nakayama et al., 2000). High sequence similarities and striking structural parallels between the phage tail structures and bacterial pyocins reveal a clear evolutionary connection between these complex molecular devices (Nakayama et al., 2000). Another phage-like structure found in bacteria including *P. aeruginosa* is a dynamic bacterial type VI secretion system (T6SS) used for translocation of virulence factors into target cells: the same mechanism the phage uses to transfer its genome to the host (Basler et al., 2012). Moreover, a recent report has shown that phage tail-like structures produced by marine bacterium *Pseudoalteromonas luteoviolacea* can trigger metamorphosis of a marine tubeworm, providing novel insights into the intricate interaction between phage, bacterium and animal (Shikuma et al., 2014).

Taken together, it is clear that phages play key roles in shaping the evolution of bacteria and vice versa. Phages not only manipulate expression of bacterial genes but also provide competitiveness for their hosts to thrive in the ecosystem as that would mutually be beneficial for both parties. Substantial structural and mechanistic analogies among bacterial puncturing devices, pyocins and secretion systems indicate an intimate evolutionary relationship between phages and their hosts.

## PHAGES IN MICROBIAL ECOSYSTEMS

Extensive *in vitro* characterization of host–phage relationships indicates short-term arms races are run between hosts and parasites (Hall et al., 2011); however, in natural habitats the interaction continues over extended time-periods. A recent study proposed that the co-evolution of phages and soil microbial communities is more likely driven by fluctuating selection dynamics, which can continue indefinitely (Gómez and Buckling, 2011). In a large ecosystem, an immune bacterial population gains benefit from the bactericidal effects of phages that act against other competitive species in the same pool and selectively enrich the ecosystem. It may therefore be better to view phage–host relationships as not merely parasitic but as mutualistic (Williams, 2013).

In an attempt to better understand phage–bacteria population dynamics, extensive metagenomic studies have been performed to characterize microbial communities in various niches, including marine, soil, and animal hosts. Metagenomic studies of human feces have demonstrated high abundance and diversity of phages (Minot et al., 2011, 2013). Barr et al. (2013) demonstrated that the abundance of phages on mucosal surfaces of epithelial cells can provide defense against bacterial infection, suggesting a plausible model for phage therapy against host mucosal infection such as foodborne infection by *S. enterica* or lung infection by *P. aeruginosa*.

As naturally designed predators to balance bacterial ecology, and at a time when multi-drug resistant bacteria are becoming widespread, phages have been identified as potential antimicrobial agents for biocontrol applications in various applications: medical therapy, agribusiness, and food safety. In the case of *S. enterica*, there have been reports of the use of phages as antimicrobial testing agents in areas ranging from food-producing animals to ready-to-eat foods (Wall et al., 2010; Guenther et al., 2012). In the case of *P. aeruginosa* phages, many reports have suggested that phages can be used to treat *P. aeruginosa* infected wounds and to disrupt the bacterial biofilm (recently reviewed in Wittebole et al., 2014). Nonetheless, it is crucial to remain aware of host–phage dynamics when developing phage therapies. The typical structure of phage–host populations in fluctuating selection dynamics is that immune bacteria are present in high abundance and are infected by wide host range phages while sensitive bacteria are much less abundant and are infected by both wide and narrow host range phages (Flores et al., 2011). Narrow host range phages are needed and must be carefully selected to ensure that the low abundance bacterial population has no change at resurgence.

## NEW RESEARCH APPROACHES IN PHAGE–HOST INTERACTION

**FIGURE 2:**
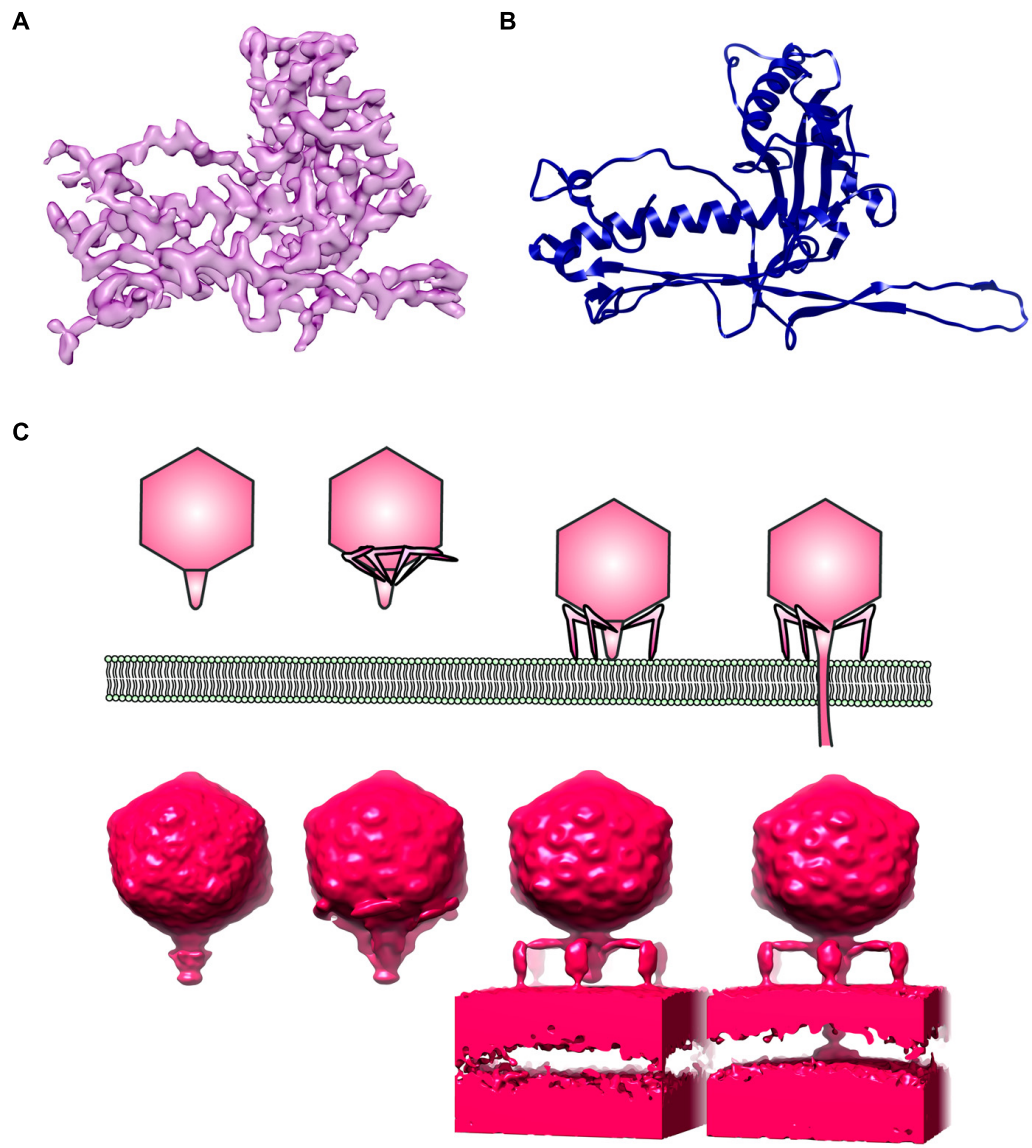
**Electron microscopy (EM) of phages. (A)** An electron density map of a major capsid protein (MCP) from *Bordetella pertussis* phage BPP1 determined by single particle reconstruction at 3.5 Angstrom resolution. **(B)** A ribbon model of BPP1 MCP (Image rendered from deposited structure, EMD-5766 and PDB ID 3J4U; Zhang et al., 2013), **(C)** a *top* cartoon shows different stages of T7 phage infection derived from electron cryotomography based on Hu et al. (2013); *bottom* shows surface rendition of electron density map generated from electron cryotomography and subtomogram averaging (Images rendered from deposited EMDB structures, EMD-5534, EMD-5535, EMD-5536, EMD-5537; Hu et al., 2013).

In terms of host–phage interaction, structural biology and microbiology are counterparts. Although several detailed structures of bacteriophages have been determined at near atomic resolution by cryoEM and image analysis (e.g., **Figures 2A,B**; Lander et al., 2012; Guo et al., 2013; Zhang et al., 2013), concern has been raised regarding poor data quality in EM of bacteriophages (Ackermann, 2014). It is also important to keep in mind that identified host–phage interactions are based on a limited number of microbial systems. It is indeed a challenge to catch the action of bacteriophages in their native niches. The invention of a small portable cryo-plunger for cryoEM that allows vitrification of environmental sample on site has opened new opportunities to merge the strengths of metagenomics and of cryoEM (Comolli et al., 2012; Luef et al., 2013). Combining metagenomics and cryogenic electron tomography (cryoET), an imaging technique suitable for visualizing the 3D organization of a pleiomorphic structure at moderate resolution, will reveal unexplored worlds within the microbial community. In fact, cryoET has been used to capture the extensive structural rearrangement of virus particles and their molecular machineries, e.g., during T7 phage infection (**Figure 2C**; Hu et al., 2013), the assembly of cyanophage (Dai et al., 2013) and the formation of virus-associated pyramids (VAPs) for the egression of archaeal virus (Daum et al., 2014).

In addition, the cryoEM 3D reconstruction of phages and virus can be vastly improved by averaging taking advantage of the icosahedral symmetry of the virus (Guo et al., 2013; Li et al., 2013; Zhang et al., 2013). Unfortunately, applying symmetry will completely mask all the unique non-symmetrical features such as base plates and tails. Of interest, a new type of extremely sensitive complementary metal oxide semiconductor (CMOS) detector has recently been developed (McMullan et al., 2009). This detector allows correction for drift and beam-induced motions of the specimen, thus images can be acquired at higher resolution. Bai et al. (2013) were able to reconstruct the 3D asymmetrical structure of ribosome at near atomic resolution using only 30,000 particles. Currently, more routine image processing and data acquisition protocols are being refined for advanced single particle analysis (Bai et al., 2013; Li et al., 2013). Combining single particle EM with biochemical experiments, 3D snapshots of bacteriophages trapped in various states can potentially be achievable at near atomic resolution.

Moreover, an electron-optical equivalent of “phase contrast” light microscopy called “phase plate” is being developed. Various prototypical versions of phase plate have been shown to improve contrast in EM significantly (Danev and Nagayama, 2011; Glaeser, 2013). Glaeser et al. (2013) showed that, with phase plate, small proteins such as individual streptavidin tetramers can be clearly distinguished in cryo. Furthermore, using phase contrast cryoEM, the 3D structure of epsilon15 bacteriophage can be resolved at nanometer resolution even with no symmetry imposed (Murata et al., 2010). Remarkably, structural details such as the tail and capsid of an individual phage can be directly delineated by electron cryo-tomographic reconstruction (Murata et al., 2010). Once these elaborate EM instruments are in place, they will enable not only a relatively routine 3D rendition of phages at high resolution using single particle analysis, but will also elucidate phage structural dynamics in the physiological environment.

Advances in metagenomics analyses and sequencing technologies have not only led to a much better appreciation of the abundance of uncultivable environmental microbial populations, but they have also enabled the monitoring of community dynamics (Breitbart and Rohwer, 2005; Edwards and Rohwer, 2005; Suttle, 2005; Rosario and Breitbart, 2011; Breitbart, 2012; Duhaime and Sullivan, 2012). However, only a handful of insights on the novel phage species can be gained through such techniques. Combining metagenomic analysis with correlative light-EM, which allows visualization of the same sample with both fluorescent and EM (Knierim et al., 2012; Luef et al., 2013; Chang et al., 2014; Schorb and Briggs, 2014), will lead to a “visual metagenomics” that will drive the genomic identification and structural characterization of novel uncultivable phages. Clearly, this is just the beginning of a new era of phage research.

## CONCLUSIONS AND PERSPECTIVES

In this review, we summarize the physical interactions between tailed phages and commonly found Gram-negative bacterial pathogens, specifically *S. enterica* and *P. aeruginosa*, which can be used as models, for other pathogens in the development of phage therapeutic strategy. The ongoing evolutionary battles between phages and bacteria continue to shape microbial communities. As the co-evolution continues, bacteria develop resistance and exploit phage machinery to fight other bacteria in order to increase environmental fitness while phages manipulate host behaviors by alteration of bacterial gene expression. Recent advances in metagenomic analysis open new doors to the vast resources of genetic diversity of viromes from various habitats, while cryoET gives much clearer pictures of how phages and hosts interact at the molecular level. Functional characterization of phages in the laboratory, combined with cryoET and subtomogram averaging, provide snapshots of different stages in phage infection. Furthermore, recent developments in direct-electron detection technology, phase plate and single particle EM provide routine protocols for determination of 3D structures of phages at near-atomic resolution. These could potentially revolutionize our understanding of the complex interplay between phages and hosts in their natural ecosystems, which is of fundamental importance in phage use in biocontrol and therapeutic strategies.

## Conflict of Interest Statement

The authors declare that the research was conducted in the absence of any commercial or financial relationships that could be construed as a potential conflict of interest.

## References

[B1] Ackermann H. W. (2014). Sad state of phage electron microscopy. Please shoot the messenger. *Microorganisms* 2 1–10 10.3390/microorganisms2010001 PMC5029504 27694773

[B2] Ackermann H. W. Prangishvili D. (2012). Prokaryote viruses studied by electron microscopy. *Arch. Virol.* 157 1843–1849 10.1007/s00705-012-1383-y 22752841

[B3] Andres D. Roske Y. Doering C. Heinemann U. Seckler R. Barbirz S. (2012). Tail morphology controls DNA release in two *Salmonella* phages with one lipopolysaccharide receptor recognition system. *Mol. Microbiol.* 83 1244–1253 10.1111/j.1365-2958.2012.08006.x 22364412

[B4] Bae H. Cho Y. (2013). Complete genome sequence of *Pseudomonas aeruginosa* podophage MPK7, which requires type IV pili for infection. *Genome Announc.* 1 e00744–e00813 10.1128/genomeA.00744-13 24115538 PMC3795208

[B5] Bai X. C. Fernandez I. S. McMullan G. Scheres S. H. W. (2013). Ribosome structures to near-atomic resolution from thirty thousand cryo-EM particles. *Elife* 2 e00461 10.7554/eLife.00461 PMC3576727 23427024

[B6] Barr J. J. Auro R. Furlan M. Whiteson K. L. Erb M. L. Pogliano J. (2013). Bacteriophage adhering to mucus provide a non-host-derived immunity. *Proc. Natl. Acad. Sci. U.S.A.* 110 10771–10776 10.1073/pnas.1305923110 23690590 PMC3696810

[B7] Basler M. Pilhofer M. Henderson G. P. Jensen G. J. Mekalanos J. J. (2012). Type VI secretion requires a dynamic contractile phage tail-like structure. *Nature* 483 182–186 10.1038/nature10846 22367545 PMC3527127

[B8] Bergh O. Borsheim K. Y. Bratbak G. Heldal M. (1989). High abundance of viruses found in aquatic environments. *Nature* 340 467–468 10.1038/340467a0 2755508

[B9] Breitbart M. (2012). Marine viruses: truth or dare. *Ann. Rev. Mar. Sci.* 4 425–448 10.1146/annurev-marine-120709-142805 22457982

[B10] Breitbart M. Hewson I. Felts B. Mahaffy J. M. Nulton J. Salamon P. Rohwer F. (2003). Bacteriophages, transposons, and plasmids: metagenomic analyses of an uncultured viral community from human feces. *J. Bacteriol.* 185 6220–6223 10.1128/JB.185.20.6220-6223.2003 14526037 PMC225035

[B11] Breitbart M. Rohwer F. (2005). Here a virus, there a virus, everywhere the same virus? *Trends Microbiol.* 13 278–284 10.1016/j.tim.2005.04.003 15936660

[B12] Casjens S. R. Gilcrease E. B. Winn-Stapley D. A. Schicklmaier P. Schmieger H. Pedulla M. L. (2005). The generalized transducing *Salmonella* bacteriophage es18: complete genome sequence and DNA packaging strategy. *J. Bacteriol.* 187 1091–1104 10.1128/JB.187.3.1091-1104.2005 15659686 PMC545730

[B13] Chang Y. W. Chen S. Tocheva E. I. Treuner-Lange A. Löbach S. Søgaard-Andersen L. (2014). Correlated cryogenic photoactivated localization microscopy and cryo-electron tomography. *Nat. Methods* 11 737–739 10.1038/nmeth.2961 24813625 PMC4081473

[B14] Choi Y. Shin H. Lee J. Ryu S. (2013). Identification and characterization of a novel flagellum-dependent *Salmonella*-infecting bacteriophage, iEPS5. *Appl. Environ. Microbiol.* 79 164829–164837 10.1128/AEM.00706-13 PMC3754727 23747700

[B15] Comolli L. R. Duarte R. Baum D. Luef B. Downing K. H. Larson D. M. (2012). A portable cryo-plunger for on-site intact cryogenic microscopy sample preparation in natural environments. *Microsc. Res. Tech.* 75 829–836 10.1002/jemt.22001 22213355 PMC4677670

[B16] Cumby N. Edwards A. M. Davidson A. R. Maxwell K. L. (2012). The bacteriophage HK97 gp15 moron element encoded a novel superinfection exclusion protein. *J. Bacteriol.* 194 5012–5019 10.1128/JB.00843-12 22797755 PMC3430355

[B17] Dai W. Fu C. Raytcheva D. Flanagan J. Khant H. A. Liu X. (2013). Visualizing virus assembly intermediates inside marine cyanobacteria. *Nature* 502 707–710 10.1038/nature12604 24107993 PMC3984937

[B18] Danev R. Nagayama K. (2011). Optimizing the phase shift and the cut-on periodicity of phase plates for TEM. *Ultramicroscopy* 111 1305–1315 10.1016/j.ultramic.2011.04.004 21864771

[B19] Daum B. Quax T. E. F. Sachse M. Mills D. J. Reimann J. Yildiz Ö. (2014). Self-assembly of the general membrane remodeling protein PVAP into sevenfold virus associated pyramids. *Proc. Natl. Acad. Sci. U.S.A.* 111 3829–3834 10.1073/pnas.1319245111 24567401 PMC3956195

[B20] Duhaime M. B. Sullivan M. B. (2012). Ocean viruses: rigorously evaluating the metagenomic sample-to-sequence pipeline. *Virology* 434 181–186 10.1016/j.virol.2012.09.036 23084423

[B21] Edwards R. A. Rohwer F. (2005). Viral metagenomics. *Nat. Rev. Microbiol.* 3 504–510 10.1038/nrmicro1163 15886693

[B22] Flores C. O. Meyer J. R. Valverde S. Farr L. Weitz J. S. (2011). Statistical structure of host–phage interactions. *Proc. Natl. Acad. Sci. U.S.A.* 108 E288–E297 10.1073/pnas.1101595108 21709225 PMC3136311

[B23] Fokine A. Rossmann M. G. (2014). Molecular architecture of tailed double-stranded DNA phages. *Bacteriophage* 4 e28281 10.104161/bact.28281 PMC3940491 24616838

[B24] Fuhrman J. A. (1999). Marine viruses and their biogeochemical and ecological effects. *Nature* 399 541–548 10.1038/21119 10376593

[B25] Glaeser R. M. (2013). Methods for imaging weak-phase objects in electron microscopy. *Rev. Sci. Instrum.* 84 111101 10.1063/1.4830355 PMC3855062 24289381

[B26] Glaeser R. M. Sassolini S. Cambie R. Jin J. Cabrini S. Schmid A. K. (2013). Minimizing electrostatic charging of an aperture used to produce in-focus phase contrast in the TEM. *Ultramicroscopy* 135 6–15 10.1016/j.ultramic.2013.05.023 23872037 PMC3818313

[B27] Gogarten J. P. Doolittle W. F. Lawrence J. G. (2002). Prokaryotic evolution in light of gene transfer. *Mol. Biol. Evol.* 19 2226–2238 10.1093/oxfordjournals.molbev.a004046 12446813

[B28] Gómez P. Buckling A. (2011). Bacteria-phage antagonistic coevolution in soil. *Science* 332 106–109 10.1126/science.1198767 21454789

[B29] Guenther S. Herzig O. Fieseler L. Klumpp J. Loessner M. J. (2012). Biocontrol of *Salmonella Typhimurium* in RTE foods with bacteriophage FO1-E2. *Int. J. Food Microbiol.* 154 66–72 10.1016/j.ijfoodmicro.2011.12.023 22244192

[B30] Guo F. Liu Z. Vago F. Ren Y. Wu W. Wright E. T. (2013). Visualization of uncorrelated, tandem symmetry mismatches in the internal genome packaging apparatus of bacteriophage T7. *Proc. Natl. Acad. Sci.* 110 6811–6816 10.1073/pnas.1215563110 23580619 PMC3637776

[B31] Hall A. R. Scanlan P. D. Morgan A. D. Buckling A. (2011). Host-parasite coevolutionary arms races give way to fluctuating selection. *Ecol. Lett.* 14 634–642 10.1111/j.1461-0248.2011.01624.x 21521436

[B32] Hanlon G. W. Denyer S. P. Olliff C. J. Ibrahim L. J. (2001). Reduction in exopolysaccharide viscosity as an aid to bacteriophage penetration through *Pseudomonas aeruginosa* biofilms. *Appl. Environ. Microbiol.* 67 2746–2753 10.1128/AEM.67.6.2746-2753.2001 11375190 PMC92934

[B33] Hendrix R. W. Smith M. C. Burns R. N. Ford M. E. Hatfull G. F. (1999). Evolutionary relationships among diverse bacteriophages and prophages: all the world’s a phage. *Proc. Natl. Acad. Sci. U.S.A.* 96 2192–2197 10.1073/pnas.96.5.2192 10051617 PMC26759

[B34] Heo Y. Chung I. Choi K. B. Lau G. W. Cho Y. (2007). Genome sequence comparison and superinfection between two related *Pseudomonas aeruginosa* phages, D3112 and MP22. *Microbiology* 153 2885–2895 10.1099/mic.0.2007/007260-0 17768233

[B35] Ho T. D. Slauch J. M. (2001). OmpC is the receptor for Gifsy-1 and Gifsy-2 bacteriophages of *Salmonella*. *J. Bacteriol.* 183 1495–1498 10.1128/JB.183.4.1495-1498.2001 11157969 PMC95030

[B36] Hosseinidoust Z. Tufenkji N. van de Ven T. G. M. (2013). Predation in homogeneous and heterogeneous phage environments affects virulence determinants of *Pseudomonas aeruginosa*. *Appl. Environ. Microbiol.* 79 2862–2871 10.1128/AEM.03817-12 23435883 PMC3623153

[B37] Hu B. Margolin W. Molineux I. J. Liu J. (2013). The bacteriophage T7 virion undergoes extensive structural remodeling during infection. *Science* 339 576–579 10.1126/science.1231887 23306440 PMC3873743

[B38] Kim M. Kim S. Park B. Ryu S. (2014). Core lipopolysaccharide-specific phage SSU5 as an auxiliary component of a phage cocktail for *Salmonella* biocontrol. *Appl. Environ. Microbiol.* 80 1026–1034 10.1128/AEM.03494-13 24271179 PMC3911222

[B39] Kim M. Ryu S. (2011). Characterization of a T5-like coliphage, SPC35, and differential development of resistance to SPC35 in *Salmonella enterica* serovar *Typhimurium* and *Escherichia coli*. *Appl. Environ. Microbiol.* 77 2042–2050 10.1128/AEM.02504-10 21257810 PMC3067339

[B40] Kim M. Ryu S. (2012). Spontaneous and transient defence against bacteriophage by phase-variable glucosylation of O-antigen in *Salmonella enterica* serovar *Typhimurium*. *Mol. Microbiol.* 86 411–425 10.1111/j.1365-2958.2012.08202.x 22928771

[B41] Kimura M. Jia Z. Nakayama N. Asakawa S. (2008). Ecology of viruses in soils: past, present and future perspectives. *Soil Sci. Plant Nutr.* 54 1–32 10.1111/j.1747-0765.2007.00197.x

[B42] Knierim B. Luef B. Wilmes P. Webb R. I. Auer M. Comolli L. R. (2012). Correlative microscopy for phylogenetic and ultrastructural characterization of microbial communities. *Environ. Microbiol. Rep.* 4 36–41 10.1111/j.1758-2229.2011.00275.x 23757227 PMC4444221

[B43] Labrie S. J. Samson J. E. Moineau S. (2010). Bacteriophage resistance mechanisms. *Nat. Rev. Microbiol.* 8 817–827 10.1038/nrmicro2315 20348932

[B44] Lander G. C. Baudoux A. C. Azam F. Potter C. S. Carragher B. Johnson J. E. (2012). Capsomer dynamics and stabilization in the T = 12 marine bacteriophage SIO-2 and its procapsid studied by CryoEM. *Structure* 20 498–503 10.1016/j.str.2012.01.007 22405008 PMC3302160

[B45] Le S. He X. Tan Y. Huang G. Zhang L. Lux R. (2013). Mapping the tail fiber as the receptor binding protein responsible for differential host specificity of *Pseudomonas aeruginosa* bacteriophages PaP1 and JG004. *PLoS ONE* 8:e68562 10.1371/journal.pone.0068562 PMC3706319 23874674

[B46] Li X. Mooney P. Zheng S. Booth C. Braunfeld M. B. Gubbens S. (2013). Electron counting and beam-induced motion correction enable near atomic resolution single particle cryoEM. *Nat. Methods* 10 584–590 10.1038/nmeth.2472 23644547 PMC3684049

[B47] Lindberg A. A. (1973). Bacteriophage receptors. *Annu. Rev. Microbiol.* 27 205–241 10.1146/annurev.mi.27.100173.001225 4584686

[B48] Luef B. Fakra S. C. Csencsits R. Wrighton K. C. Williams K. H. Wilkins M. J. (2013). Iron-reducing bacteria accumulate ferric oxyhydroxide nanoparticle aggregates that may support planktonic growth. *ISME J.* 7 338–350 10.1038/ismej.2012.103 23038172 PMC3554402

[B49] Marsh P. Wellington E. M. (1994). Phage-host interaction in soil. *FEMS Microbiol. Ecol.* 15 99–108 10.1111/j.1574-6941.1994.tb00234.x

[B50] Marti R. Zurfluh K. Hagens S. Pianezzi J. Klumpp J. Loessner M. J. (2013). Long tail fibres of the novel broad-host-range T-even bacteriophage S16 specifically recognize *Salmonella* OmpC. *Mol. Microbiol.* 87 818–834 10.1111/mmi.12134 23289425

[B51] McMullan G. Faruqi A. R. Henderson R. Guerrini N. Turchetta R. van Hoften G. (2009). Experimental observation of the improvement in MTF from backthinning a CMOS direct electron detector. *Ultramicroscopy* 109 1144–1147 10.1016/j.ultramic.2009.05.005 19541421 PMC2937214

[B52] Mills S. Shanahan F. Stanton C. Hill C. Coffey A. Ross R. P. (2013). Movers and shakers: influence of bacteriophages in shaping the mammalian gut microbiota. *Gut Microbes* 4 4–16 10.4161/gmic.22371 23022738 PMC3555884

[B53] Minot S. Bryson A. Chehoud C. Wu G. D. Lewis J. D. Bushman F. D. (2013). Rapid evolution of the human gut virome. *Proc. Natl. Acad. Sci. U.S.A.* 110 12450–12455 10.1073/pnas.1300833110 23836644 PMC3725073

[B54] Minot S. Sinha R. Chen J. Li H. Keilbaugh S. A. Wu G. D. (2011). The human gut virome: inter-individual variation and dynamic response to diet. *Genome Res.* 21 1616–1625 10.1101/gr.122705.111 21880779 PMC3202279

[B55] Murata K. Liu X. Danev R. Jakana J. Schmid M. F. King J. (2010). Zernike phase contrast cryo-electron microscopy and tomography for structure determination at nanmeter and subnanometer resolutions. *Structure* 18 903–912 10.1016/j.str.2010.06.006 20696391 PMC2925294

[B56] Nakayama K. Takashima K. Ishihara H. Shinomiya T. Kageyama M. Kanaya S. (2000). The R-type pyocin of *Pseudomonas aeruginosa* is related to P2 phage, and the F-type is related to lambda phage. *Mol. Microbiol.* 38 213–231 10.1046/j.1365-2958.2000.02135.x 11069649

[B57] Novick R. P. Christie G. E. Penades J. R. (2010). The phage-related chromosomal islands of gram-positive bacteria. *Nat. Rev. Microbiol.* 8 541–551 10.1038/nrmicro2393 20634809 PMC3522866

[B58] Pickard D. Toribio A. L. Petty N. K. de Tonder A. Yu L. Goulding D. (2010). A conserved acetyl esterase domain targets diverse bacteriophage to the Vi capsular receptor of *Salmonella enterica* serovar *Typhi*. *J. Bacteriol.* 192 5746–5754 10.1128/JB.00659-10 20817773 PMC2953684

[B59] Ricci V. Piddock L. J. V. (2010). Exploiting the role of TolC in pathogenicity: identification of a bacteriophage for eradication of *Salmonella* serovars from poultry. *Appl. Environ. Microbiol.* 76 1704–1706 10.1128/AEM.02681-09 20080996 PMC2832399

[B60] Rosario K. Breitbart M. (2011). Exploring the viral world through metagenomics. *Curr. Opin. Virol.* 1 289–297 10.1016/j.coviro.2011.06.004 22440785

[B61] Samson J. E. Magadan A. H. Sabri M. Moineau S. (2013). Revenge of the phages: defeating bacterial defences. *Nat. Rev. Microbiol.* 11 675–687 10.1038/nrmicro3096 23979432

[B62] Schorb M. Briggs J. A. (2014). Correlated cryo-fluorescence and cryo-electron microscopy with high spatial precision and improved sensitivity. *Ultramicroscopy* 143 24–32 10.1016/j.ultramic.2013.10.015 24275379 PMC5472196

[B63] Shikuma N. J. Pilhofer M. Weiss G. L. Hadfield M. G. Jensen G. J. Newman D. K. (2014). Marine tapeworm metamorphosis induced by arrays of bacterial phage tail-like structures. *Science* 343 529–533 10.1126/science.1246794 24407482 PMC4949041

[B64] Shin H. Lee J. Kim H. Choi Y. Heu S. Ryu S. (2012). Receptor diversity and host interaction of bacteriophages infecting *Salmonella enterica* serovar *Typhimurium*. *PLoS ONE* 7:e43392 10.1371/journal.pone.0043392 PMC3424200 22927964

[B65] Susskind M. M. Botstein D. Wright A. (1974a). Superinfection exclusion by P22 prophage in lysogens of *Salmonella typhimurium*. III. Failure of superinfecting phage DNA to enter sieA+ lysogens. *Virology* 62 350–366 10.1016/0042-6822(74)90398-5 4610992

[B66] Susskind M. M. Wright A. Botstein D. (1974b). Superinfection exclusion by P22 prophage in lysogens of *Salmonella typhimurium*. IV. Genetics and physiology of sieB exclusion. *Virology* 62 367–384 10.1016/0042-6822(74)90399-7 4610993

[B67] Suttle C. A. (2005). Viruses in the sea. *Nature* 437 356–361 10.1038/nature04160 16163346

[B68] Suttle C. A. (2007). Marine viruses-major players in the global ecosystem. *Nat. Rev. Microbiol.* 5 801–812 10.1038/nrmicro1750 17853907

[B69] Temple G. S. Ayling P. D. Wilkinson S. G. (1986). Isolation and characterization of a lipopolysaccharide-specific bacteriophage of *Pseudomonas aeruginosa*. *Microbios* 45 81–91 10.1099/0022-1317-33-1-99 3086673

[B70] Thingstad T. F. Lignell R. (1997). Theoretical models for the control of bacterial growth rate, abundance, diversity and carbon demand. *Aquat. Microb. Ecol.* 13 19–27 10.3354/ame013019

[B71] Veesler D. Cambillau C. (2011). A common evolutionary origin for tailed-bacteriophage functional modules and bacterial machineries. *Microbiol. Mol. Biol. Rev.* 75 423–433 10.1128/MMBR.00014-11 21885679 PMC3165541

[B72] Wall S. K. Zhang J. Rostagno M. H. Ebner P. D. (2010). Phage therapy to reduce preprocessing *Salmonella* infections in market-weight swine. *Appl. Environ. Microbiol.* 76 48–53 10.1128/AEM.00785-09 19854929 PMC2798657

[B73] Webber M. A. Bailey A. M. Blair J. M. A. Morgan E. Stevens M. P. Hinton J. C. D. (2009). The global consequence of disruption of the AcrAB-TolC eﬄux pump in *Salmonella enterica* includes reduced expression of SPI-1 and other attributes required to infect the host. *J. Bacteriol.* 191 4276–4285 10.1128/JB.00363-09 19411325 PMC2698494

[B74] Williams H. T. P. (2013). Phage-induced diversification improves host evolvability. *BMC Evol. Biol.* 13:17 10.1186/1471-2148-13-17 PMC3605116 23339571

[B75] Wittebole X. De Roock S. Opal S. M. (2014). A historical overview of bacteriophage therapy as an alternative to antibiotics for the treatment of bacterial pathogens. *Virulence* 5 225–235 10.4161/viru.25991 PMC3916379 23973944

[B76] Zhang X. Guo H. Jin L. Czornyj E. Hodes A. Hui W. H. (2013). A new topology of the HK97-like fold revealed in *Bordetella* bacteriophage by cryoEM at 3.5 Å resolution. *Elife* 2 e01299 10.7554/elife.01299 PMC3863775 24347545

